# Phenotype-stratified treatment response in obese atrial fibrillation: Post-hoc cluster analysis of the PRAGUE-25 randomized trial

**DOI:** 10.1016/j.ijcha.2026.101931

**Published:** 2026-04-19

**Authors:** Ivan Ranic, Ladislav Stanke, Otakar Jiravsky, Dalibor Herman, Tomas Roubicek, Stepan Havranek, Jan Chovancik, Veronika Bulkova, Martin Matoulek, Vladimir Tuka, Jana Hozmanova, Marek Hozman, Adam Latinak, Jan Pidhorodecky, Milan Dusik, Jan Simek, Bogna Jiravska-Godula, Jan Alexander Mohr, Zuzana Hejdukova, Klara Benesova, Jiri Plasek, Pavel Osmancik

**Affiliations:** a Department of Cardiology, Agel Hospital Trinec-Podlesi, Trinec, Czech Republic; b Faculty of Medicine, University of Ostrava, Ostrava, Czech Republic; c Department of Psychology, Faculty of Arts, Palacky University Olomouc, Czech Republic; d Cardiocenter, Third Faculty of Medicine, Charles University Prague and University Hospital Kralovske Vinohrady, Prague, Czech Republic; e Department of Cardiology, Regional Hospital, Liberec, Czech Republic; f Cardiocenter, Second Internal Clinic – Cardiology and Angiology, Charles University, General Faculty Hospital, Prague, Czech Republic; g Department of Cardiology, Neuron Medical Center, Hospital Brno, Brno, Czech Republic; h Institute of Biostatistics and Analyses, Faculty of Medicine, Masaryk University, Brno, Czech Republic; i Department of Physiology, Faculty of Medicine, Masaryk University, Brno, Czech Republic

**Keywords:** Atrial fibrillation, Obesity, Catheter ablation, Cluster analysis, Phenotype, Treatment response

## Abstract

**Background:**

The PRAGUE-25 trial demonstrated catheter ablation (CA) superiority over lifestyle modification plus antiarrhythmic drugs (LFM + AAD) in obese patients with atrial fibrillation (AF). However, obese AF patients represent a heterogeneous population with varying pathophysiological substrates. We hypothesized that distinct patient phenotypes may exhibit differential treatment responses.

**Methods:**

This post-hoc analysis applied hierarchical cluster analysis (Ward’s D2 method, Euclidean distance) to 122 PRAGUE-25 patients with complete data using six delta (12-month minus baseline) echocardiographic and metabolic variables (Δ-LAVI, Δ-LVEDD, Δ-NT-proBNP, Δ-triglycerides, Δ-leukocytes, Δ-platelets). Treatment effects within each phenotype were compared using Fisher’s exact test and odds ratios with 95% confidence intervals.

**Results:**

Three distinct phenotypes were identified: Metabolic (n = 20, 16%), characterized by highest triglycerides with minimal structural remodeling; Intermediate Remodeling (n = 67, 55%), distinguished by largest LV chamber with lowest inflammatory burden; and Advanced Neurohormonal/Inflammatory (n = 35, 29%), exhibiting highest NT-proBNP with elevated inflammatory markers. While cluster membership did not predict overall AF freedom (χ^2^ = 3.45, p = 0.178), CA superiority was statistically significant only in the Intermediate phenotype (OR 3.98, 95% CI: 1.01–15.57, p = 0.047; AF freedom 88.5% CA vs 65.9% LFM + AAD). In the Metabolic and Advanced Neurohormonal/Inflammatory phenotypes (45% of patients), no statistically significant treatment difference was observed; however, wide confidence intervals preclude conclusions of treatment equivalence in these underpowered subgroups. These findings appear to be primarily driven by the Intermediate Remodeling phenotype.

**Conclusions:**

These hypothesis-generating findings suggest phenotype-dependent treatment response heterogeneity in obese atrial fibrillation. As cluster membership can only be determined retrospectively, prospective validation using baseline predictor models is required before clinical application.

## 1 Introduction

Atrial fibrillation (AF) and obesity represent converging epidemics, with obesity being one of the strongest modifiable AF risk factors [1], [2], [3]. The relationship is pathophysiologically complex, involving structural remodeling, inflammation, and metabolic dysfunction [4], [5]. This mechanistic heterogeneity suggests obese AF patients may not represent a homogeneous population amenable to uniform treatment strategies.

Current therapeutic approaches include both catheter ablation (CA) and lifestyle modification (including weight reduction, increased physical activity, and alcohol reduction) as effective strategies for rhythm control [6], [7]. The LEGACY trial established that sustained weight loss exceeding 10% achieves a six-fold increase in arrhythmia-free survival [8]. Catheter ablation provides the most effective rhythm control strategy including obese AF patients, though AF-freedom in obese patients has been inferior to non-obese patients [9], [10]. However, conflicting evidence exists; cryoballoon pulmonary vein isolation has demonstrated non-inferior outcomes in obese versus non-obese patients in prospective series, with comparable arrhythmia recurrence rates despite increased procedural complexity [11], [12]. Whether distinct phenotypes within the obese AF population respond differentially to these approaches remains unanswered.

The PRAGUE-25 trial randomized 203 obese patients with symptomatic AF to either catheter ablation or comprehensive lifestyle modification plus antiarrhythmic drugs (LFM + AAD) [13]. At 12-month follow-up, catheter ablation demonstrated superiority for the primary endpoint of AF freedom (73.0% vs 34.6%; OR 2.97, p < 0.001), while LFM + AAD arm achieved significant metabolic benefits including greater weight loss and improved glycemic control. However, the trial's overall results may mask phenotype-specific treatment effects within this heterogeneous population. Understanding which patients benefit most from each treatment approach could inform personalized therapeutic strategies.

We therefore performed a post-hoc cluster analysis of PRAGUE-25 data with the following objectives: (1) to identify clinically meaningful patient phenotypes using unsupervised hierarchical cluster analysis based on delta parameters capturing structural, metabolic, and inflammatory changes during follow-up; (2) to characterize phenotype-specific clinical profiles; and (3) to explore phenotype-treatment interactions. Given the post-hoc, exploratory design, these findings are hypothesis-generating and require prospective validation.

## 2 Methods

### 2.1 Study design and population

This post-hoc exploratory analysis examined data from the PRAGUE-25 trial (NCT04011800), a prospective, randomized, open-label trial comparing catheter ablation versus lifestyle modification plus antiarrhythmic drugs in obese patients with AF [13], [14]. The parent trial enrolled 212 patients between May 2021 and November 2023 at five Czech centers. The study was approved by the Ethics Committee of each participating center, and all patients signed informed consent prior to the enrolment. Nine patients withdrew consent, leaving 203 patients for primary analysis with 1:1 randomization to LFM + AAD versus CA. The primary endpoint was freedom from AF at 12 months assessed by 7-day Holter monitoring. Outpatient controls were scheduled every 3 months during the first year, and every 6 months later on. Seven-day Holter monitoring was performed every 3 months starting on the day of the procedure (or on the day of the start of full LFM program, both approx. 3 months after randomization) during the first year of the study and every 6 months later on. This post-hoc analysis was conducted using de-identified data from the parent trial. Patients were analyzed according to their original randomization assignment (intention-to-treat principle). Of the 203 ITT patients, 122 (60%) had complete data for all six clustering variables at both timepoints and were included in this analysis (56 from CA arm, 66 from LFM + AAD arm, reflecting proportions similar to the parent trial).

### 2.2 Analysis objectives

This post-hoc analysis followed a two-stage approach. First, unsupervised hierarchical cluster analysis using delta (change-score) parameters was performed to identify whether distinct patient phenotypes exist based on differential treatment response patterns—that is, how patients' structural, metabolic, and inflammatory parameters changed during follow-up. Delta-based clustering was chosen specifically to capture response heterogeneity rather than baseline disease severity alone. Second, following cluster identification, we characterized these phenotypes by comparing baseline demographics, echocardiographic parameters, and laboratory values across clusters, and assessed whether treatment efficacy differed by phenotype.

### 2.3 Endpoint definition

The primary endpoint for this subanalysis was freedom from atrial fibrillation, atrial flutter, or atrial tachycardia (AF/AFL/AT) at the scheduled 12-month visit, defined as the absence of AF/AFL/AT documentation at the final follow-up assessment (point-in-time endpoint). This differs from the primary endpoint of the parent PRAGUE-25 trial, which applied a cumulative 12-month definition: any documented AF/AFL/AT episode > 30 s at any of four scheduled Holter assessments (M3, M6, M9, M12) or at an emergency visit constituted treatment failure. The point-in-time endpoint was selected for this subanalysis because the clustering variables are also assessed at 12 months, providing temporal alignment between phenotype characterisation and outcome measurement. As a consequence of this endpoint definition, AF-freedom rates in the present subanalysis are higher than those reported in the parent trial, particularly in the LFM + AAD arm, in which patients with early recurrences who achieved sinus rhythm by 12 months are classified as successes here but failures in the parent trial. This distinction is methodologically intentional and does not indicate a clinically distinct population. AF burden (percentage of time in AF) was analysed as a secondary endpoint. Within-phenotype treatment effects (CA vs LFM + AAD) were compared to explore phenotype-treatment interactions.

Importantly, the primary (visit-based) and sensitivity (Holter-based) endpoints are methodologically independent assessments performed at the same M12 timepoint. The primary endpoint reflects clinical documentation of AF/AFL/AT recurrence at the scheduled outpatient visit — based on patient-reported symptoms, interim medical records, and spot ECG — and is recorded by the treating physician as a binary yes/no determination. The sensitivity endpoint reflects automated detection of any AF/AFL/AT episode exceeding 30 s on the accompanying 7-day continuous Holter recording. The Holter-based criterion is therefore structurally stricter: it additionally captures asymptomatic paroxysmal AF that is invisible to clinical visit assessment alone. This distinction directly explains the directionally larger CA effect sizes observed in the sensitivity analysis — LFM + AAD patients who appear in sinus rhythm at the outpatient visit may nonetheless carry residual paroxysmal AF burden detectable only on continuous monitoring.

### 2.4 Clustering variables

For the present cluster analysis, complete data were required for all six clustering variables at both baseline and 12-month follow-up. This yielded a final analytical sample of 122 patients (60% of 203). The 81 excluded patients (40%) had missing echocardiographic (mostly left atrial volume index) or laboratory data at least at one timepoint. These assessments were non-obligatory in the parent PRAGUE-25 protocol.

To assess potential selection bias, included patients (n = 122) were compared with excluded patients (n = 81) across all available baseline characteristics. No statistically significant differences were identified in age (59.8 ± 8.3 vs 60.2 ± 9.4 years, p = 0.728), body mass index (35.1 ± 3.1 vs 34.6 ± 2.9 kg/m^2^, p = 0.227), CHA_2_DS_2_-VASc score (2.0 ± 1.2 vs 2.0 ± 1.3, p = 0.853), sex (67.2% vs 70.4% male, p = 0.749), AF type (59.8% vs 49.4% paroxysmal, p = 0.186), treatment allocation (45.9% vs 54.3% catheter ablation, p = 0.302), or 12-month AF-freedom (77.9% vs 80.2%, p = 0.818). A full comparison is provided in Supplementary Table S8. These results do not support systematic selection bias; however, the numerically higher AF-freedom in excluded patients (80.2% vs 77.9%) represents a directional trend that cannot be entirely excluded as a source of bias and is acknowledged as a limitation.

Six delta variables (12-month value minus baseline value) were selected a priori to capture pathophysiologically relevant domains in obese AF: Δ-LAVI (left atrial volume index, mL/m^2^) — marker of structural atrial remodeling; Δ-LVEDD (left ventricular end-diastolic diameter, mm) — marker of ventricular remodeling; Δ-NT-proBNP (pg/mL) — cardiac stress and heart failure biomarker; Δ-Triglycerides (mmol/L) — metabolic status marker; Δ-Leukocytes (×10^9^/L) — inflammatory burden marker; Δ-Platelets (×10^9^/L) — thrombotic and inflammatory marker.

Variables were standardized using z-score transformation prior to clustering. Unlike prior AF phenotyping studies using baseline characteristics [15], [16], we employed change-score parameters to capture treatment response patterns rather than baseline disease state alone. To the best of our knowledge, this represents the first application of delta-based clustering for AF treatment response phenotyping.

### 2.5 Clustering Algorithm

Agglomerative hierarchical clustering was performed using Ward's minimum variance method (D2) with Euclidean distance [17]. Ward's method was selected because it minimizes total within-cluster variance at each merging step, producing compact clusters with minimal intra-cluster heterogeneity—properties well-suited for identifying distinct patient phenotypes [18]. The three-cluster solution was selected based on dendrogram inspection showing clear separation at this level, clinical interpretability of resulting phenotypes, and statistical discrimination between clusters. Analysis was performed using the snowCluster module in jamovi statistical software.

### 2.6 Statistical analysis

Baseline characteristics were compared across phenotypes using Welch's one-way ANOVA for continuous variables (to account for potential heterogeneity of variances across groups) with Bonferroni-corrected post-hoc pairwise comparisons (Supplementary Tables S1–S3). Categorical variables were compared using Fisher's exact test given the small sample sizes in some cells.

Within-cluster treatment effects were analyzed using Fisher's exact test. Odds ratios with 95% confidence intervals were calculated for AF freedom comparing catheter ablation versus LFM + AAD within each phenotype. The association between cluster membership and the primary outcome was assessed using chi-square test. Mantel-Haenszel pooled odds ratio was calculated to estimate overall treatment effect controlling for cluster membership.

Cluster structure was validated using multinomial logistic regression to assess the ability of clustering variables and demographic factors to predict cluster membership. Model fit was evaluated using McFadden's pseudo-R2 and likelihood ratio chi-square test. Welch's ANOVA results and post-hoc pairwise comparisons are provided in Supplementary Tables S4–S5. Normality of continuous variables was assessed using Shapiro-Wilk test, and homogeneity of variances was evaluated using Levene's test.

A formal test of treatment-by-cluster interaction was performed using binary logistic regression with cluster membership (dummy-coded, Cluster 1 as reference), treatment arm, and two treatment × cluster interaction terms. Cluster-specific treatment odds ratios with 95% confidence intervals were derived from linear combinations of regression coefficients using the delta method. Model fit was compared between the main-effects model and the interaction model using the likelihood ratio test. All analyses were performed using Python 3 (statsmodels 0.14).

Two pre-specified sensitivity analyses were performed. First, cluster-specific treatment effects were re-estimated using a Holter-based 12-month endpoint (any AF/AFL/AT on the 12-month Holter recording) in place of the primary scheduled-visit endpoint. Second, the Cluster 2 odds ratio was validated by bootstrap resampling (5,000 iterations, seed 42) to confirm stability of the point estimate and provide a non-parametric confidence interval. Full results are provided in Supplementary Table S9.

## 3 Results

### 3.1 Phenotype identification

**Fig. 1:**
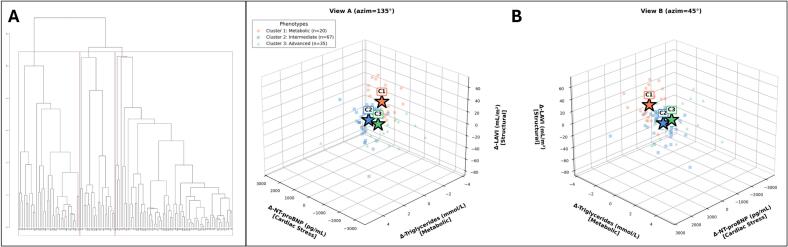
Hierarchical Clustering Dendrogram and Three-Dimensional Phenotype Visualization − Dendrogram derived from agglomerative hierarchical clustering (Ward's D2 method, Euclidean distance) of 122 patients based on six delta variables (12-month minus baseline): ΔLAVI, ΔLVEDD, ΔNT-proBNP, Δtriglycerides, Δleukocytes, and Δplatelets. The horizontal axis represents individual patients; the vertical axis quantifies inter-individual dissimilarity (squared Euclidean distance). Red vertical lines denote the three-cluster partition selected based on dendrogram topology and clinical interpretability. Vertical branch lengths reflect the magnitude of between-cluster divergence, with the initial high-level bifurcation separating two principal branches and a subsequent split yielding three phenotypically distinct groups. − Three-dimensional scatter plots from complementary viewing angles displaying patient distribution across the three primary clustering dimensions: Δ-triglycerides, Δ-NT-proBNP, and Δ-LAVI. Orange circles = Cluster 1, Metabolic phenotype (n = 20); Blue squares = Cluster 2, Intermediate Remodeling phenotype (n = 67); Green triangles = Cluster 3, Advanced Neurohormonal/Inflammatory phenotype (n = 35). Stars (★) indicate cluster centroids. Abbreviations: LAVI = left atrial volume index; LVEDD = left ventricular end-diastolic diameter; NT-proBNP = N-terminal pro-B-type natriuretic peptide. (For interpretation of the references to colour in this figure legend, the reader is referred to the web version of this article.)

**Fig. 2:**
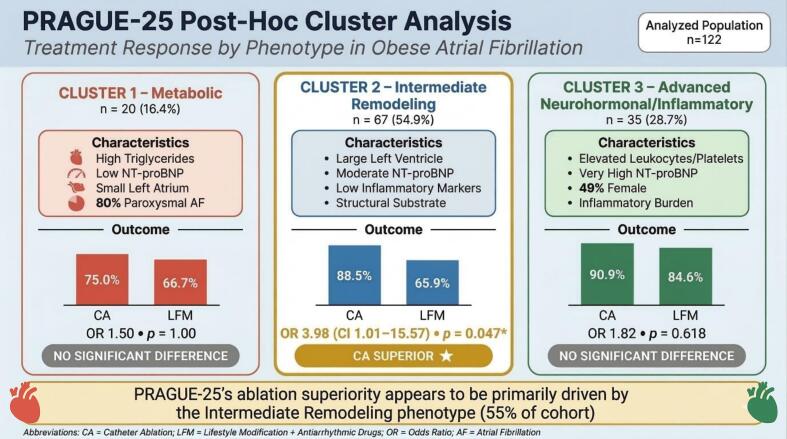
Treatment Response by Phenotype (Graphical Abstract) Summary illustration depicting the three identified phenotypes with their key baseline characteristics and treatment responses among 122 analyzed patients. NT-proBNP is listed as the second characteristic in each panel for consistent cross-phenotype comparison. The Intermediate Remodeling phenotype (Cluster 2, center, n = 67, 55%) demonstrates statistically significant catheter ablation superiority (AF freedom: 88.5% CA vs 65.9% LFM + AAD; OR 3.98, 95% CI: 1.01–15.57, p = 0.047), characterized by structural substrate with low inflammatory burden. The Metabolic phenotype (Cluster 1, left, n = 20, 16%) and Advanced Neurohormonal/Inflammatory phenotype (Cluster 3, right, n = 35, 29%) show no statistically significant treatment difference; wide confidence intervals in these smaller subgroups preclude conclusions of treatment equivalence. These findings suggest PRAGUE-25′s overall ablation superiority appears to be primarily driven by the Intermediate Remodeling phenotype. The Advanced Neurohormonal/Inflammatory label (Cluster 3) reflects the dominant pathophysiology of elevated NT-proBNP and inflammatory markers, not structural remodeling. Abbreviations: CA = catheter ablation; LFM = lifestyle modification plus antiarrhythmic drugs; OR = odds ratio.

Hierarchical cluster analysis of 122 patients with complete data identified three distinct phenotypes (Fig. 1). Baseline characteristics of the cluster analysis population are presented in Table 1, with extended demographics, echocardiographic parameters, and laboratory values in Supplementary Tables S1–S3 for all three clusters (Fig. 2).

**Table 1:** Baseline Characteristics by Phenotype.

Variable	Cluster 1 (n = 20)	Cluster 2 (n = 67)	Cluster 3 (n = 35)	p-value
*Demographics*				
Age, years	59.0 ± 7.9	58.8 ± 8.3	62.1 ± 8.2	0.143
Male sex, n (%)	15 (75%)	49 (73%)	18 (51%)	0.062
BMI, kg/m^2^	35.3 ± 3.5	35.2 ± 3.2	34.9 ± 2.9	0.857
Paroxysmal AF, n (%)	**16 (80%)**	39 (58%)	18 (51%)	0.073
CHA_2_DS_2_-VASc score	2.0 ± 1.2	1.9 ± 1.2	2.3 ± 1.3	0.352

*Echocardiographic Parameters*				
LAVI, mL/m^2^	**38.8 ± 9.4**	44.6 ± 18.4	42.9 ± 13.4	0.358
LVEDD, mm	49.2 ± 6.0	**54.2 ± 10.4**	50.7 ± 4.7	0.030*
LVEF, %	62.4 ± 5.8	61.0 ± 6.4	60.1 ± 7.6	0.476

*Laboratory Values*				
NT-proBNP, pg/mL	**234 ± 269**	403 ± 357	**911 ± 931**	<0.001***
Triglycerides, mmol/L	**2.87 ± 1.24**	**1.56 ± 0.68**	1.85 ± 1.23	<0.001***
Leukocytes, ×10^9^/L	7.22 ± 1.49	**6.95 ± 1.91**	**8.22 ± 1.35**	0.002**
Platelets, ×10^9^/L	226 ± 40	232 ± 60	**261 ± 55**	0.022*

Values are mean ± SD or n (%). Bold values indicate defining phenotype features as described in Results; bold p-values indicate statistical significance. *p < 0.05, **p < 0.01, ***p < 0.001. LAVI = left atrial volume index; LVEDD = left ventricular end-diastolic diameter; LVEF = left ventricular ejection fraction. Extended baseline data available in Supplementary Tables S1–S3.

### 3.2 Cluster 1: Metabolic AF phenotype (n = 20, 16.4%)

This phenotype was defined by the largest reduction in triglycerides during follow-up (Δ-TG − 1.35 ± 1.03 mmol/L, p < 0.001), with minimal changes in structural parameters (Δ-LAVI + 19.5 ± 27.6 mL/m^2^; Δ-LVEDD + 7.2 ± 18.8 mm) and stable NT-proBNP (Δ − 1.5 ± 221 pg/mL). Baseline values of clustering variables showed prominent metabolic derangement (TG 2.87 ± 1.24 mmol/L) with the smallest left atrium (LAVI 38.8 ± 9.4 mL/m^2^) and lowest NT-proBNP (234 ± 269 pg/mL). Demographics: 75% male, age 59.0 ± 7.9 years, BMI 35.3 ± 3.5 kg/m^2^, 80% paroxysmal/15% persistent/5% long-standing persistent AF. Treatment allocation: 40% CA (n = 8), 60% LFM + AAD (n = 12).

### 3.3 Cluster 2: Intermediate Remodeling phenotype (n = 67, 54.9%)

This phenotype was defined by stable structural and metabolic parameters during follow-up: minimal change in left atrial size (Δ-LAVI − 1.6 ± 16.9 mL/m^2^), stable ventricular dimensions (Δ-LVEDD − 1.0 ± 10.5 mm), stable NT-proBNP (Δ − 24 ± 458 pg/mL), and unchanged triglycerides (Δ-TG 0.00 ± 0.59 mmol/L), with the lowest inflammatory marker changes. Baseline values showed the largest LV chamber (LVEDD 54.2 ± 10.4 mm), moderate NT-proBNP (403 ± 357 pg/mL), lowest leukocytes (6.95 ± 1.91 × 10^9^/L), and favorable metabolic profile (TG 1.56 ± 0.68 mmol/L). Demographics: 73% male, age 58.8 ± 8.3 years, BMI 35.2 ± 3.2 kg/m^2^, 58% paroxysmal/34% persistent/8% long-standing persistent AF. Treatment allocation: 39% CA, 61% LFM + AAD.

### 3.4 Cluster 3: Advanced Neurohormonal/Inflammatory phenotype (n = 35, 28.7%)

This phenotype was defined by the greatest improvement in neurohormonal markers during follow-up (Δ-NT-proBNP − 611 ± 845 pg/mL, p < 0.001 vs other clusters), with elevated baseline inflammatory burden (Δ-leukocytes, Δ-platelets among highest). Baseline values showed marked neurohormonal activation (NT-proBNP 911 ± 931 pg/mL), elevated leukocytes (8.22 ± 1.35 × 10^9^/L), and highest platelets (261 ± 55 × 10^9^/L). Demographics: 49% female (vs 25–27% in other clusters), age 62.1 ± 8.2 years, BMI 34.9 ± 2.9 kg/m^2^, 49% persistent AF (p = 0.028). Treatment allocation: 63% CA, 37% LFM + AAD.

### 3.5 Cluster validation

The cluster structure was internally validated using multinomial logistic regression (overall model: χ^2^ = 61.2, df = 10, p < 0.001; McFadden's R2 = 0.255). Of greater biological interest, sex emerged as a significant predictor of cluster membership (χ2 = 9.50, p = 0.009) despite not being included among the clustering variables, with the Advanced Neurohormonal/Inflammatory phenotype (Cluster 3) showing female predominance (49% vs 25–27% in other clusters). Welch's ANOVA results for key clustering variables are provided in Supplementary Table S4, with Tukey post-hoc pairwise comparisons in Supplementary Table S5. Phenotype labels were assigned based on the most clinically discriminating baseline characteristics of each cluster, providing clinically intuitive nomenclature. Cluster membership was determined by delta parameter patterns during follow-up; the labels therefore reflect the baseline substrate context, not the clustering variables themselves.

### 3.6 Treatment response by phenotype

Cluster membership did not significantly predict overall AF freedom at 12 months (χ^2^ = 3.45, p = 0.178). Overall success rates were 70.0%, 74.6%, and 88.6% in Clusters 1, 2, and 3, respectively. Differential treatment response was observed within phenotypes (Table 2).

**Table 2:** Treatment Outcomes by Phenotype — AF Freedom at 12 Months.

**Phenotype**	**CA Success**	**LFM + AAD Success**	**OR (95% CI)**	**p-value**
Cluster 1 — Metabolic (n = 20)	75.0% (6/8)	66.7% (8/12)	1.50 (0.20–11.1)	1.000
**Cluster 2 — Intermediate Remodeling (n = 67)**	**88.5% (23/26)**	65.9% (27/41)	**3.98 (1.01**–**15.57)**	0.047*
Cluster 3 — Advanced Neurohormonal/Inflammatory (n = 35)	90.9% (20/22)	84.6% (11/13)	1.82 (0.22–14.8)	0.618
*Cluster-outcome association (χ^2^ test)*				0.178

*p < 0.05 (Fisher's exact test for within-cluster comparisons). CA = catheter ablation; LFM + AAD = lifestyle modification plus antiarrhythmic drugs; OR = odds ratio; CI = confidence interval. Cluster 2 (highlighted) is the only phenotype demonstrating statistically significant ablation superiority. Absolute difference in Cluster 2: 22.6%. Absence of statistical significance in Clusters 1 and 3 does not confirm treatment equivalence; wide confidence intervals reflect limited power (n = 20 and n = 35 respectively) and a clinically meaningful catheter ablation advantage cannot be excluded.

The Intermediate Remodeling phenotype (Cluster 2) was the only group demonstrating statistically significant catheter ablation superiority. AF freedom was achieved in 88.5% (23/26) of ablation patients versus 65.9% (27/41) of LFM + AAD patients (OR 3.98, 95% CI: 1.01–15.57, p = 0.047), representing a 22.6% absolute difference. This phenotype, comprising 55% of the analytical sample, appears to drive the overall PRAGUE-25 result.

In the Metabolic (Cluster 1) and Advanced Neurohormonal/Inflammatory (Cluster 3) phenotypes, no statistically significant difference in AF freedom between treatment arms was observed. In Cluster 1, AF freedom was 75.0% (6/8) with ablation versus 66.7% (8/12) with LFM + AAD (OR 1.50, 95% CI: 0.20–11.1, p = 1.000). In Cluster 3, AF freedom was 90.9% (20/22) with ablation versus 84.6% (11/13) with LFM + AAD (OR 1.82, 95% CI: 0.22–14.8, p = 0.618). Wide confidence intervals in both subgroups (n = 20 and n = 35) preclude conclusions of treatment equivalence; a clinically meaningful catheter ablation advantage cannot be excluded. These two phenotypes represent 45% of the patient population.

Mantel-Haenszel pooled odds ratio controlling for cluster membership was 2.71, suggesting a consistent overall treatment effect across phenotypes, though the magnitude of benefit varies substantially.

### 3.7 Formal test of Treatment-by-Cluster interaction

A binary logistic regression model incorporating treatment arm, cluster membership, and treatment × cluster interaction terms was fitted to the cluster subpopulation (n = 122). The likelihood ratio test comparing the interaction model with the main-effects model showed no statistically significant treatment-by-cluster interaction (χ^2^ = 0.78, df = 2, p = 0.677). This result is consistent with insufficient statistical power for formal heterogeneity testing at the available sample size and does not preclude a clinically meaningful differential treatment effect. Achieving adequate power for this test would require approximately 300–500 patients with complete follow-up data. Cluster-specific treatment odds ratios derived from the interaction model were consistent with the primary Fisher's exact test results: Cluster 1 OR 1.50 (95% CI 0.20–11.09, p = 0.691); Cluster 2 OR 3.98 (95% CI 1.01–15.57, p = 0.048); Cluster 3 OR 1.82 (95% CI 0.22–14.75, p = 0.576). The marginal difference in p-values for Cluster 2 (Fisher's exact p = 0.047 versus logistic regression p = 0.048) reflects the distinct distributional assumptions of the two tests and is not clinically meaningful.

### 3.8 Sensitivity analyses

Bootstrap resampling of the Cluster 2 subgroup (n = 67; 5,000 iterations) confirmed robustness of the primary point estimate. The bootstrapped median OR was 4.06 (95% CI: 1.17–17.68), closely reproducing the Fisher's exact result (OR 3.98, 95% CI 1.01–15.57). The lower bound of the bootstrap confidence interval (1.17) does not cross unity, providing non-parametric confirmation of the finding.

When the primary scheduled-visit endpoint was replaced by a Holter-based 12-month endpoint, treatment effects were directionally consistent. Cluster 2 results were replicated and strengthened (OR 4.86, 95% CI 1.57–15.1, p = 0.006). Notably, Cluster 3 also reached statistical significance on this endpoint definition (OR 7.39, 95% CI 1.23–44.4, p = 0.020), suggesting that the observed absence of statistical significance in Cluster 3 on the primary endpoint may be endpoint-dependent rather than a robust biological finding. Full sensitivity analysis results are provided in Supplementary Table S9.

## 4 Discussion

### 4.1 Principal findings

In this post-hoc analysis of the PRAGUE-25 trial, hierarchical cluster analysis based on delta parameters identified three distinct patient phenotypes among obese AF patients. While cluster membership did not predict overall AF freedom, we observed significant phenotype-dependent variation in treatment response. The Intermediate Remodeling phenotype, characterized by structural substrate with minimal metabolic and inflammatory burden, was the only group demonstrating statistically significant catheter ablation superiority (OR 3.98, p = 0.047). This phenotype comprises 55% of patients and appears to drive the overall PRAGUE-25 result. In the Metabolic and Advanced Neurohormonal/Inflammatory phenotypes (45% of patients), no statistically significant treatment difference was observed; however, wide confidence intervals and the Holter-based sensitivity analysis (Cluster 3 OR 7.39, p = 0.020) indicate that conclusions of treatment equivalence cannot be drawn from these underpowered subgroup comparisons.

### 4.2 Mechanistic Interpretation

The Intermediate Remodeling phenotype is characterized by structural cardiac substrate (largest LVEDD, 54.2 mm) with minimal metabolic and inflammatory burden (lowest triglycerides, lowest leukocytes). This phenotype may represent the optimum target for catheter ablation, where structural remodeling has created anatomically targetable arrhythmogenic substrate, but without diffuse fibrosis or systemic inflammation that might render ablation less effective [19], [20]. The moderate NT-proBNP elevation (403 pg/mL) suggests cardiac stress that remains compensated — optimal conditions for successful ablation.

The absence of a significant treatment difference in Metabolic and Advanced Neurohormonal/Inflammatory phenotypes does not represent ablation 'failure'. Rather, it may reflect that lifestyle modification is highly effective in these phenotypes because they possess substantial modifiable substrate. In the Metabolic phenotype, the highest triglycerides and metabolic dysfunction respond to lifestyle intervention. In the Advanced Neurohormonal/Inflammatory phenotype, elevated inflammatory markers and neurohormonal activation may respond to the anti-inflammatory and neurohormonal benefits of weight loss and exercise [8], [21]. These observations are hypothesis-generating and require prospective validation.

### 4.3 The combined therapy hypothesis

This hypothesis is supported by emerging evidence. The ARREST-AF study demonstrated that aggressive risk factor management improved catheter ablation outcomes [22]. The LEGACY trial showed that weight loss exceeding 10% was associated with a six-fold increase in arrhythmia-free survival.8 The REVERSE-AF study confirmed that weight loss reduced AF burden independent of ablation [21]. Our analysis provides a conceptual framework for understanding these findings—patients with modifiable substrate (Metabolic and Advanced phenotypes) may derive greater benefit from lifestyle modification in addition to ablation, while those with predominantly structural substrate (Intermediate phenotype) require ablation but may have limited rhythm control benefit from intensive lifestyle programs.

### 4.4 Clinical Implications and prospective Translation

The present findings are exploratory and hypothesis-generating. Because cluster membership is defined by 12-month delta parameters, it cannot be determined prospectively at the time of treatment decision. The observed baseline differences between phenotypes — particularly in triglycerides, NT-proBNP, and inflammatory markers — are hypothesis-generating observations that identify potential baseline predictors of phenotype membership. Such a prediction model would require independent development and external validation before any clinical application could be considered. The current findings do not support changes to clinical practice and should not be interpreted as justification for phenotype-based treatment selection outside of a research context.

### 4.5 Prospective prediction and future Directions

Because phenotypes are defined by treatment-induced changes, cluster membership can only be determined retrospectively, precluding direct application for prospective therapeutic decisions. However, the identified phenotypes differ significantly in baseline characteristics that may serve as proxy predictors. The Metabolic phenotype exhibited highest triglycerides (2.87 mmol/L), smallest left atrium, and lowest NT-proBNP with predominantly paroxysmal AF. The Advanced Neurohormonal/Inflammatory phenotype showed marked neurohormonal activation (NT-proBNP 911 pg/mL), elevated inflammatory markers, and female predominance. The Intermediate phenotype occupied a middle position without these extremes.

These differences are hypothesis-generating observations. A key future research priority is the prospective development and independent validation of a baseline prediction tool — potentially incorporating triglycerides, NT-proBNP, inflammatory markers, and AF type — that could identify patients likely to show differential treatment response. Such validation falls outside the scope of the current post-hoc analysis.

Several additional research priorities emerge. A randomized trial comparing ablation alone versus ablation plus intensive lifestyle modification in patients with high metabolic or inflammatory baseline profiles would test the combined therapy hypothesis. Cardiac MRI with late gadolinium enhancement could characterize fibrosis patterns across phenotypes. Novel pharmacotherapy may be phenotype-specific: the Metabolic phenotype may benefit from GLP-1 receptor agonists achieving substantial weight reduction [23], [24]; the Advanced Neurohormonal/Inflammatory phenotype with elevated NT-proBNP may benefit from SGLT2 inhibitors [25] or anti-inflammatory approaches including colchicine [26].

## 5 Limitations

### 5.1 Several limitations warrant consideration

First, this was a post-hoc exploratory analysis with inherent risk of false-positive findings from multiple comparisons; results should be interpreted as hypothesis-generating only.

Second, the Metabolic phenotype (Cluster 1, n = 20) and Advanced Neurohormonal/Inflammatory phenotype (Cluster 3, n = 35) have limited statistical power, and the absence of statistically significant treatment differences in these clusters should not be interpreted as evidence of treatment equivalence. Wide confidence intervals (Cluster 1: OR 1.50, 95% CI 0.20–11.1; Cluster 3: OR 1.82, 95% CI 0.22–14.8) reflect inadequate power rather than confirmed equivalence.

Third, the 40% exclusion rate due to missing echocardiographic or laboratory data introduces potential selection bias. A formal comparison of included (n = 122) versus excluded (n = 81) patients showed no statistically significant differences across all key characteristics (all p > 0.05; Supplementary Table S8). Nevertheless, the numerically higher AF-freedom in excluded patients (80.2% vs 77.9%) represents a directional trend that cannot be entirely excluded as a source of bias.

Fourth, the imbalanced treatment allocation in Cluster 3 (63% CA vs 37% LFM + AAD) results in small denominators for the LFM + AAD subgroup, limiting statistical power and potentially influencing cluster characterisation.

Fifth, delta-based clustering captures biological change trajectories that are partly influenced by the assigned treatment. Although treatment allocation was statistically balanced across Clusters 1 and 2 (CA 40% and 39%, respectively; χ^2^ = 5.69, p = 0.058), and within-cluster delta variables showed no statistically significant treatment-arm differences (all p > 0.05), a complete separation of treatment-induced from substrate-driven biological change is not possible with this design. This is particularly relevant for Cluster 3, where catheter ablation was numerically more prevalent (63%).

Sixth, the formal test of treatment-by-cluster interaction was not statistically significant (χ^2^ = 0.78, df = 2, p = 0.677), reflecting insufficient power for heterogeneity testing at n = 122. A minimum of approximately 300–500 patients with complete follow-up data would be required. The current findings therefore constitute a signal warranting prospective investigation rather than a confirmed treatment-effect modifier.

Seventh, the primary endpoint (point-in-time 12-month AF-freedom) differs from the cumulative endpoint used in the parent PRAGUE-25 trial, resulting in higher apparent AF-freedom rates, particularly in the LFM + AAD arm. While this choice aligns temporally with the clustering variables and is methodologically coherent, readers should interpret AF-freedom rates in the context of this distinction.

Eighth, the sensitivity analysis using a Holter-based 12-month endpoint showed statistically significant treatment differences in Cluster 3 (OR 7.39, p = 0.020) in addition to Cluster 2, suggesting that the absence of statistical significance in Cluster 3 on the primary endpoint may be endpoint-dependent. This uncertainty underscores the exploratory nature of all cluster-specific treatment comparisons.

Ninth, the 12-month follow-up may not capture long-term treatment durability differences between phenotypes.

Tenth, cluster membership is determined retrospectively based on 12-month follow-up data and cannot be applied prospectively to individual patients. Although significant baseline differences in triglycerides, NT-proBNP, inflammatory markers, and AF type were identified across phenotypes, these observations are hypothesis-generating and serve as candidate predictors only; no validated baseline classification tool currently exists.

Eleventh, the analysis derives from five Czech referral centres enrolling patients meeting specific eligibility criteria (symptomatic AF, BMI ≥ 30  kg/m^2^, age 18–65 years). Findings may not generalise to broader AF-obesity populations differing in ethnicity, healthcare system, co-morbidity burden, or access to structured lifestyle modification programmes.

Twelfth, the three-cluster solution has not been validated in an independent external cohort. Internal cluster validation (multinomial logistic regression, McFadden’s R2 = 0.255) confirms within-sample discrimination but cannot substitute for prospective external replication. Validation studies using independent obese AF datasets are required before the phenotype classification can be considered reproducible.

## 6 Conclusions

In this post-hoc cluster analysis of the PRAGUE-25 trial, we identified three distinct phenotypes among obese AF patients with different dominant substrates and differential treatment responses. The Intermediate Remodeling phenotype (55% of patients) appears to drive PRAGUE-25′s overall ablation superiority — characterized by structural substrate with minimal metabolic and inflammatory targets, catheter ablation significantly outperformed lifestyle modification in this group (OR 3.98, p = 0.047). In the Metabolic and Advanced Neurohormonal/Inflammatory phenotypes (45% of patients), no statistically significant treatment difference was observed; however, wide confidence intervals preclude conclusions of treatment equivalence, and the Holter-based sensitivity analysis suggests a potential catheter ablation advantage in Cluster 3 that the primary analysis was underpowered to detect. These hypothesis-generating findings suggest phenotype-dependent treatment response heterogeneity in obese AF. Prospective validation using baseline predictor models is required before clinical application.

## Author Contributions

Ivan Ranic, Ladislav Stanke and Otakar Jiravsky conceived and designed the post-hoc cluster analysis, performed the statistical analyses, interpreted the data, and drafted the manuscript. Pavel Osmancik designed and led the parent PRAGUE-25 trial, supervised the current analysis, and critically revised the manuscript. Tomas Roubicek, Stepan Havranek, Jan Chovancik, Veronika Bulkova, Dalibor Herman, Martin Matoulek, Vladimir Tuka contributed to patient recruitment and data acquisition and critically revised the manuscript. Jan Alexander Mohr contributed to the statistical methodology, validated the cluster analysis approach, and critically revised the manuscript. Klara Benesova contributed to data management and statistical analysis. Jiri Plasek critically revised the manuscript. All authors approved the final version.

## Funding Support

This work was supported by the Ministry of Health of the Czech Republic, grant nr. NU21-02–00388, and by the Charles University Research program “Cooperatio – Cardiovascular Science” and by Vzdelavaci a vyzkumny institut AGEL o.p.s. with grant nr. INT2025003.

## CRediT authorship contribution statement

**Ivan Ranic:** Writing – original draft, Methodology, Investigation, Data curation, Conceptualization. **Ladislav Stanke:** Writing – original draft, Visualization, Formal analysis, Data curation, Conceptualization. **Otakar Jiravsky:** Writing – original draft, Visualization, Methodology, Data curation, Conceptualization. **Dalibor Herman:** Writing – review & editing, Investigation. **Tomas Roubicek:** Writing – review & editing, Investigation. **Stepan Havranek:** Writing – review & editing, Project administration, Investigation. **Jan Chovancik:** Writing – review & editing, Methodology, Investigation. **Veronika Bulkova:** Writing – review & editing, Project administration, Investigation. **Martin Matoulek:** Writing – review & editing, Software, Methodology, Investigation. **Vladimir Tuka:** Writing – review & editing, Methodology, Investigation. **Jana Hozmanova:** Writing – review & editing, Project administration, Investigation. **Marek Hozman:** Writing – review & editing, Investigation. **Adam Latinak:** Investigation. **Jan Pidhorodecky:** Investigation. **Milan Dusik:** Writing – review & editing, Methodology, Investigation. **Jan Simek:** Writing – review & editing, Investigation, Conceptualization. **Bogna Jiravska-Godula:** Writing – review & editing, Methodology, Investigation. **Jan Alexander Mohr:** Validation, Methodology, Data curation, Conceptualization. **Zuzana Hejdukova:** Writing – review & editing, Methodology, Investigation. **Klara Benesova:** Validation, Methodology, Data curation, Conceptualization. **Jiri Plasek:** Writing – review & editing, Validation. **Pavel Osmancik:** Writing – review & editing, Formal analysis, Data curation.

## Declaration of competing interest

The authors declare the following financial interests/personal relationships which may be considered as potential competing interests: Pavel Osmancik reports administrative support, article publishing charges, statistical analysis, and writing assistance were provided by Ministry of Health of the Czech Republic, grant nr. NU21-02–00388. Pavel Osmancik reports administrative support, article publishing charges, statistical analysis, and writing assistance were provided by Charles University Research program. Otakar Jiravsky reports article publishing charges and statistical analysis were provided by Vzdelavaci a vyzkumny institut AGEL o.p.s. with grant nr. INT2025003. If there are other authors, they declare that they have no known competing financial interests or personal relationships that could have appeared to influence the work reported in this paper.

## Data Availability

The data underlying this analysis are available from the corresponding author upon reasonable request and subject to appropriate data sharing agreements.

## Appendix A Supplementary data

Supplementary Data 1 CONSORT flow diagram (Figure S1) and extended baseline, follow-up, statistical, medication, and sensitivity-analysis data (Tables S1–S9) for the three identified phenotypes.
