# Supplementary material for: Phenotype-stratified treatment response in obese atrial fibrillation: Post-hoc cluster analysis of the PRAGUE-25 randomized trial
Source: Int J Cardiol Heart Vasc. 2026 Apr 19;64:101931. doi: 10.1016/j.ijcha.2026.101931 (PMC13122199; doi:10.1016/j.ijcha.2026.101931)
Supplement: Supplementary Data 1 — CONSORT flow diagram (Figure S1) and extended baseline, follow-up, statistical, medication, and sensitivity-analysis data (Tables S1–S9) for the three identified phenotypes. [file mmc1.docx]

Supplementary Appendix to:

**Phenotype-Stratified Treatment Response in Obese Atrial Fibrillation:**

**Post-Hoc Cluster Analysis of the PRAGUE-25 Randomized Trial**

*Manuscript IJCHA-D-26-00078*

**LIST OF SUPPLEMENTARY MATERIALS**

**Supplementary Figure S1.** CONSORT Flow Diagram for Post-Hoc Cluster Analysis

**Supplementary Table S1.** Extended Baseline Demographics and Clinical Characteristics

**Supplementary Table S2.** Extended Echocardiographic Parameters

**Supplementary Table S3.** Extended Laboratory Values

**Supplementary Table S4.** Welch’s ANOVA Results for Key Clustering Variables

**Supplementary Table S5.** Tukey Post-Hoc Pairwise Comparisons

**Supplementary Table S6.** Changes from Baseline to 12 Months by Phenotype

**Supplementary Table S7.** Baseline Medication Use by Phenotype

**Supplementary Table S8.** Comparison of Baseline Characteristics: Included vs. Excluded Patients

**Supplementary Table S9.** Sensitivity Analyses: Treatment Effects by Phenotype Using Alternative Endpoint Definitions

**Supplementary Figure S1.** CONSORT Flow Diagram for Post-Hoc Cluster Analysis

**
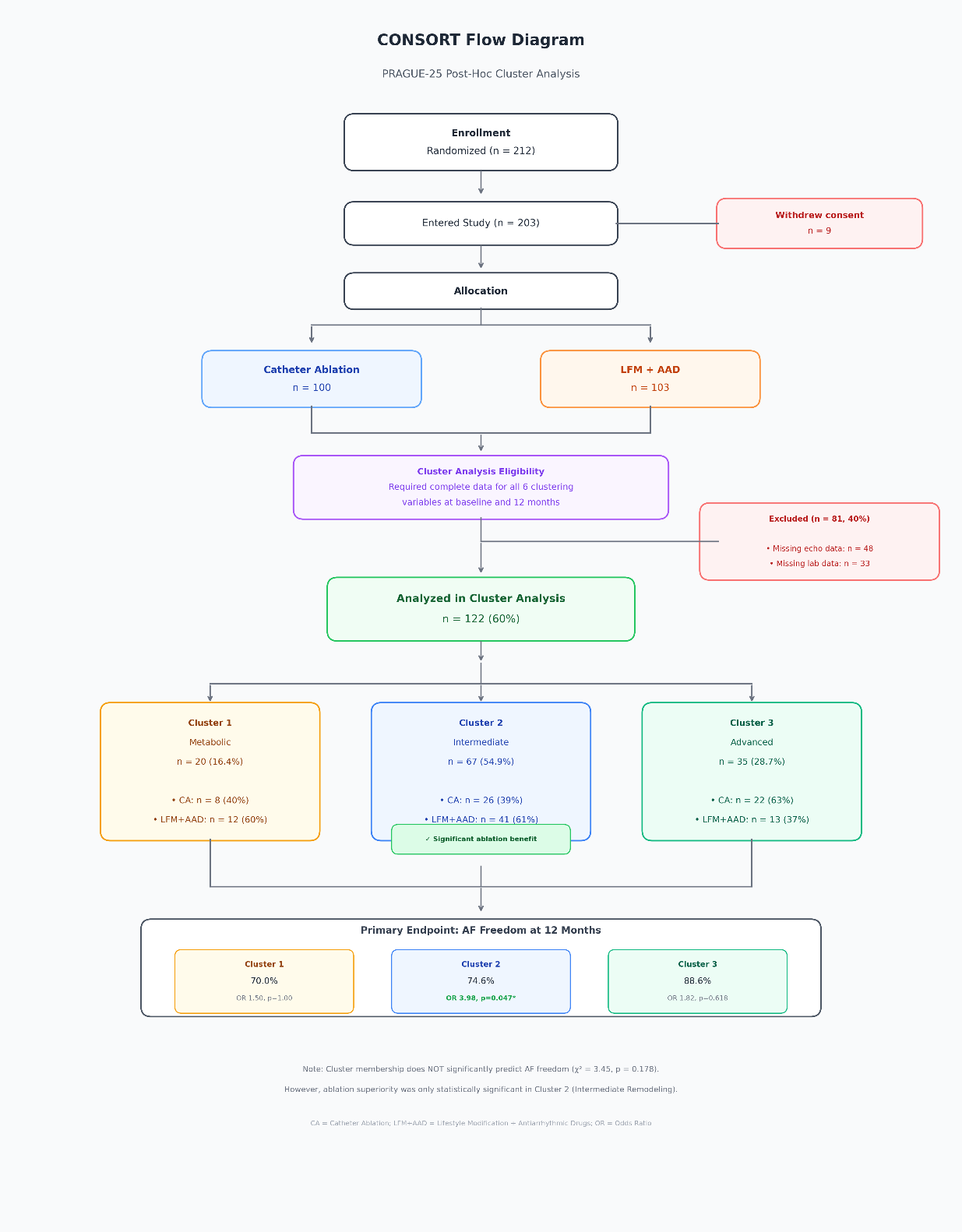
**

**Figure S1.** Of 212 patients randomised in the parent PRAGUE-25 trial, 9 withdrew consent, leaving 203 patients in the intention-to-treat population (catheter ablation [CA] arm n=100; lifestyle modification plus antiarrhythmic drugs [LFM+AAD] arm n=103). Of these, 122 patients (60%) had complete data for all six clustering variables (Δ-LAVI, Δ-LVEDD, Δ-NT-proBNP, Δ-triglycerides, Δ-leukocytes, Δ-platelets) at both baseline and 12-month follow-up and were included in the cluster analysis. The 81 excluded patients (40%) had missing echocardiographic data (n=48) or missing laboratory data (n=33) at one or both timepoints; these measurements were non-obligatory in the parent PRAGUE-25 protocol. A comparison of baseline characteristics between included and excluded patients showed no statistically significant differences across any key variable (Supplementary Table S8). The 122 analysed patients were assigned to three phenotypes by Ward's D2 hierarchical cluster analysis: *Cluster 1 — Metabolic* (n=20, 16.4%; CA n=8, LFM+AAD n=12); Cluster 2 — Intermediate Remodeling (n=67, 54.9%; CA n=26, LFM+AAD n=41); Cluster 3 — Advanced Neurohormonal/Inflammatory (n=35, 28.7%; CA n=22, LFM+AAD n=13).

**Supplementary Table S1. Extended Baseline Demographics and Clinical Characteristics**

| **Variable** | **Cluster 1 (n=20)** | **Cluster 2 (n=67)** | **Cluster 3 (n=35)** | **p-value** |
| --- | --- | --- | --- | --- |
| Age, years | 59.0 ± 7.9 | 58.8 ± 8.3 | 62.1 ± 8.2 | 0.143 |
| Male sex, n (%) | 15 (75%) | 49 (73%) | **18 (51%)** | 0.062 |
| BMI, kg/m² | 35.3 ± 3.5 | 35.2 ± 3.2 | 34.9 ± 2.9 | 0.857 |
| Paroxysmal AF, n (%) | **16 (80%)** | 39 (58%) | 18 (51%) | 0.073 |
| Persistent AF, n (%) | 3 (15%) | 23 (34%) | **17 (49%)** | **0.028* |
| Diabetes mellitus, n (%) | **6 (30%)** | 18 (27%) | 5 (14%) | 0.269 |
| Hypertension, n (%) | 18 (90%) | 53 (79%) | 30 (86%) | 0.449 |
| CHA₂DS₂-VASc score | 2.0 ± 1.2 | 1.9 ± 1.2 | 2.3 ± 1.3 | 0.352 |
| Ablation group, n (%) | 8 (40%) | 26 (39%) | **22 (63%)** | 0.052 |

*Bold indicates the most extreme value for that variable across the three clusters. p-values from Welch's one-way ANOVA (continuous variables) or Fisher's exact test (categorical variables). * p<0.05. Abbreviations: BMI = body mass index; AF = atrial fibrillation; CHA₂DS₂-VASc = Congestive heart failure, Hypertension, Age ≥75, Diabetes mellitus, Stroke/TIA, Vascular disease, Age 65–74, Sex category score.*

**Supplementary Table S2. Extended Echocardiographic Parameters**

| **Variable** | **Cluster 1 (n=20)** | **Cluster 2 (n=67)** | **Cluster 3 (n=35)** | **p-value** |
| --- | --- | --- | --- | --- |
| LAVI, mL/m² | **38.8 ± 9.4** | 44.6 ± 18.4 | 42.9 ± 13.4 | 0.358 |
| LVEDD, mm | 49.2 ± 6.0 | **54.2 ± 10.4** | 50.7 ± 4.7 | **0.030* |
| LVEF, % | 62.4 ± 5.8 | 61.0 ± 6.4 | 60.1 ± 7.6 | 0.476 |
| LA dimension, mm | 43.4 ± 6.3 | 45.8 ± 5.5 | 45.6 ± 6.3 | 0.249 |
| Septal thickness, mm | 12.2 ± 5.3 | 11.0 ± 2.2 | 10.9 ± 1.4 | 0.234 |
| TR gradient, mmHg | 13.1 ± 11.3 | 18.7 ± 12.1 | **23.3 ± 9.8** | **0.034* |
| E/e’ ratio | 9.9 ± 2.8 | 9.2 ± 3.2 | 10.2 ± 3.8 | 0.347 |

*Bold indicates the most extreme value for that variable across the three clusters. p-values from Welch's one-way ANOVA. * p<0.05. Abbreviations: LAVI = left atrial volume index; LVEDD = left ventricular end-diastolic diameter; LVEF = left ventricular ejection fraction; LA = left atrium; TR = tricuspid regurgitation; E/e' = early transmitral velocity to early diastolic mitral annular velocity ratio.*

**Supplementary Table S3. Extended Laboratory Values**

| **Variable** | **Cluster 1 (n=20)** | **Cluster 2 (n=67)** | **Cluster 3 (n=35)** | **p-value** |
| --- | --- | --- | --- | --- |
| NT-proBNP, pg/mL | **234 ± 269** | 403 ± 357 | **911 ± 931** | **<0.001*** |
| Triglycerides, mmol/L | **2.87 ± 1.24** | **1.56 ± 0.68** | 1.85 ± 1.23 | **<0.001*** |
| Leukocytes, ×10⁹/L | 7.22 ± 1.49 | **6.95 ± 1.91** | **8.22 ± 1.35** | **0.002** |
| Platelets, ×10⁹/L | 226 ± 40 | 232 ± 60 | **261 ± 55** | **0.022* |
| Fasting glucose, mmol/L | **6.48 ± 1.51** | 5.99 ± 1.17 | 5.96 ± 1.35 | 0.292 |
| HbA1c, mmol/mol | 38.3 ± 12.4 | 39.3 ± 7.4 | 42.5 ± 10.8 | 0.180 |
| Total cholesterol, mmol/L | 4.95 ± 1.18 | 4.52 ± 1.19 | 4.40 ± 1.10 | 0.228 |
| LDL cholesterol, mmol/L | 2.91 ± 1.24 | 2.70 ± 1.02 | 2.41 ± 0.96 | 0.202 |
| HDL cholesterol, mmol/L | 1.17 ± 0.29 | 1.24 ± 0.31 | 1.31 ± 0.32 | 0.250 |
| CRP, mg/L | 4.2 ± 3.1 | 3.6 ± 2.6 | **5.8 ± 4.9** | **0.017* |
| Hemoglobin, g/L | 153 ± 12 | 151 ± 14 | 146 ± 18 | 0.143 |
| Creatinine, µmol/L | 91.3 ± 18.9 | 87.2 ± 19.3 | 91.0 ± 25.5 | 0.603 |

*Bold indicates the most extreme value(s) for that variable across the three clusters. p-values from Welch's one-way ANOVA. * p<0.05. Abbreviations: NT-proBNP = N-terminal pro-B-type natriuretic peptide; LDL = low-density lipoprotein; HDL = high-density lipoprotein; CRP = C-reactive protein; HbA1c = glycated haemoglobin.*

**Supplementary Table S4. Welch’s ANOVA Results for Key Clustering Variables**

| **Variable** | **F** | **df1** | **df2** | **p-value** |
| --- | --- | --- | --- | --- |
| NT-proBNP (pg/mL) | 8.65 | 2 | 50.8 | **<0.001*** |
| Triglycerides (mmol/L) | 10.40 | 2 | 39.1 | **<0.001*** |
| Leukocytes (×10⁹/L) | 8.09 | 2 | 53.4 | **<0.001*** |
| Platelets (×10⁹/L) | 4.37 | 2 | 56.7 | **0.017* |
| LVEDD (mm) | 4.02 | 2 | 55.4 | **0.023* |
| TR gradient (mmHg) | 3.92 | 2 | 32.4 | **0.030* |

*Welch's ANOVA was used to account for potential heterogeneity of variances across clusters. df1 = numerator degrees of freedom (between groups = 2); df2 = denominator degrees of freedom (Welch-adjusted, approximated). Bold indicates the largest F-statistic. * p<0.05. Post-hoc pairwise comparisons with Bonferroni correction are provided in Supplementary Table S5. Abbreviations: LVEDD = left ventricular end-diastolic diameter; TR = tricuspid regurgitation.*

**Supplementary Table S5. Tukey Post-Hoc Pairwise Comparisons**

| **Variable** | **Comparison** | **Mean Difference** | **p-value** |
| --- | --- | --- | --- |
| NT-proBNP (pg/mL) |  |  |  |
|  | Cluster 1 vs 2 | −169 | 0.481 |
|  | Cluster 1 vs 3 | −677 | **<0.001*** |
|  | Cluster 2 vs 3 | −508 | **<0.001*** |
| Triglycerides (mmol/L) |  |  |  |
|  | Cluster 1 vs 2 | +1.31 | **<0.001*** |
|  | Cluster 1 vs 3 | +1.02 | **<0.001*** |
|  | Cluster 2 vs 3 | −0.29 | 0.323 |
| Leukocytes (×10⁹/L) |  |  |  |
|  | Cluster 1 vs 2 | +0.27 | 0.808 |
|  | Cluster 1 vs 3 | −1.01 | 0.093 |
|  | Cluster 2 vs 3 | −1.28 | **0.001** |
| Platelets (×10⁹/L) |  |  |  |
|  | Cluster 1 vs 2 | −5.62 | 0.917 |
|  | Cluster 1 vs 3 | −35.3 | 0.066 |
|  | Cluster 2 vs 3 | −29.6 | **0.032* |

*Pairwise comparisons between cluster means following Welch's one-way ANOVA, Bonferroni-corrected. Mean difference is expressed in the original unit of the variable (positive = Cluster 1 or 2 > comparator). Bold indicates statistically significant differences. * p<0.05. Abbreviations: NT-proBNP = N-terminal pro-B-type natriuretic peptide.*

**Supplementary Table S6. Changes from Baseline to 12 Months by Phenotype**

| **Variable** | **Cluster 1 (n=20)** | **Cluster 2 (n=67)** | **Cluster 3 (n=35)** | **p-value** |
| --- | --- | --- | --- | --- |
| Δ Weight, kg | −3.54 ± 6.73 | −3.14 ± 7.71 | −3.28 ± 6.05 | 0.976 |
| Δ NT-proBNP, pg/mL | −1.5 ± 221 | −24 ± 458 | **−611 ± 845** | **<0.001*** |
| Δ Triglycerides, mmol/L | **−1.35 ± 1.03** | −0.00 ± 0.59 | −0.05 ± 1.01 | **<0.001*** |
| Δ Total cholesterol, mmol/L | **−0.65 ± 1.03** | −0.13 ± 0.89 | +0.06 ± 0.72 | **0.016* |
| Δ LAVI, mL/m² | **+19.5 ± 27.6** | −1.6 ± 16.9 | −2.6 ± 16.0 | **<0.001*** |
| Δ LVEDD, mm | **+7.2 ± 18.8** | −1.0 ± 10.5 | +1.0 ± 4.4 | **0.019* |
| Δ LVEF, % | −0.3 ± 6.6 | −1.6 ± 10.5 | +0.4 ± 6.8 | 0.553 |
| Δ HbA1c, mmol/mol | +2.3 ± 12.4 | −3.0 ± 11.3 | −2.6 ± 22.6 | 0.422 |
| Δ Peak VO₂, mL/kg/min | 0.0 ± 3.9 | 0.0 ± 4.1 | +0.5 ± 3.8 | 0.873 |
| Δ CRP, mg/L | −0.7 ± 2.3 | +0.9 ± 4.2 | −1.1 ± 4.0 | **0.034* |
| Δ AF burden, % | −8.7 ± 46.0 | −10.2 ± 36.8 | −29.0 ± 42.9 | 0.097 |
| Δ AFEQT score | +8.1 ± 18.7 | +15.7 ± 20.0 | +14.3 ± 15.8 | 0.314 |

*Delta (Δ) values = 12-month measurement minus baseline measurement. Bold indicates the most extreme change across clusters for that variable. p-values from Welch's one-way ANOVA. * p<0.05. Abbreviations: LAVI = left atrial volume index; LVEDD = left ventricular end-diastolic diameter; LVEF = left ventricular ejection fraction; NT-proBNP = N-terminal pro-B-type natriuretic peptide; VO₂ = oxygen consumption; CRP = C-reactive protein; AF = atrial fibrillation; AFEQT = Atrial Fibrillation Effect on QualiTy-of-life.*

**Supplementary Table S7. Baseline Medication Use by Phenotype**

| **Medication** | **Cluster 1 (n=20)** | **Cluster 2 (n=67)** | **Cluster 3 (n=35)** | **p-value** |
| --- | --- | --- | --- | --- |
| ***Antiarrhythmic Drugs*** | | | | |
| Any antiarrhythmic, % | 66% | 54% | 60% | 0.527 |
| Propafenone, % | 45% | 37% | 29% | NS |
| Amiodarone, % | 15% | 19% | 24% | NS |
| ***Beta-Blockers*** | | | | |
| Any beta-blocker, % | 75% | 78% | 80% | 0.903 |
| Metoprolol, % | **40%** | 16% | **34%** | **0.038* |
| Bisoprolol, % | 30% | **51%** | 40% | NS |
| ***Anticoagulation*** | | | | |
| Any anticoagulation, % | 89% | 93% | 95% | 0.669 |
| NOACs, % | 90% | 94% | 97% | NS |
| Aspirin, % | 0% | 3% | 3% | NS |

*Bold indicates a statistically significant or notable inter-cluster difference for that medication class. p-values from Fisher's exact test. * p<0.05. NS = not significant (p≥0.05); exact p-values not reported for non-significant pairwise items. Abbreviations: NOACs = non-vitamin K oral anticoagulants.*

**Supplementary Table S8. Comparison of Baseline Characteristics: Included vs. Excluded Patients (n=122 vs. n=81)**

| **Variable** | **Included (n=122)** | **Excluded (n=81)** | **p-value** |
| --- | --- | --- | --- |
| Age, years | 59.8 ± 8.3 | 60.2 ± 9.4 | 0.728 |
| BMI, kg/m² | 35.1 ± 3.1 | 34.6 ± 2.9 | 0.227 |
| CHA₂DS₂-VASc score | 2.0 ± 1.2 | 2.0 ± 1.3 | 0.853 |
| Male sex, n (%) | 82 (67.2%) | 57 (70.4%) | 0.749 |
| Paroxysmal AF, n (%) | 73 (59.8%) | 40 (49.4%) | 0.186 |
| Catheter ablation allocation, n (%) | 56 (45.9%) | 44 (54.3%) | 0.302 |
| 12-month AF freedom, n (%) | 95 (77.9%) | 65 (80.2%)¹ | 0.818 |

*p-values from independent-samples t-test (continuous variables) or Fisher's exact test (categorical variables). No statistically significant differences were identified across any characteristic (all p>0.05), supporting the absence of systematic selection bias. Bold is not applicable in this two-group comparison. * p<0.05. ¹ 12-month AF freedom defined using the point-in-time endpoint (rhythm status at the scheduled 12-month visit only), consistent with the primary analysis in the present paper. This differs from the cumulative 12-month endpoint of the parent PRAGUE-25 trial, in which any documented AF/AFL/AT episode at any of four Holter assessments (M3, M6, M9, M12) constitutes treatment failure; as a result, AF-freedom rates in this paper are higher than those of the parent trial, particularly in the LFM+AAD arm. Abbreviations: BMI = body mass index; AF = atrial fibrillation; CHA₂DS₂-VASc = see Table S1.*

**Supplementary Table S9. Sensitivity Analyses: Treatment Effects by Phenotype Using Alternative Endpoint Definitions**

**Panel A: Holter-Based 12-Month Endpoint (Any AF/AFL/AT on M12 Holter Recording)**

| **Phenotype** | **CA AF-free (%)** | **LFM AF-free (%)** | **OR (95% CI)** | **p-value** |
| --- | --- | --- | --- | --- |
| Cluster 1 — Metabolic (n=20) | 75.0 | 41.7 | 4.20 (0.55–32.1) | 0.197 |
| **Cluster 2 — Intermediate Remodeling (n=67)** | **80.8** | **46.3** | **4.86 (1.57–15.1)** | **0.006** |
| Cluster 3 — Advanced Neurohormonal/Inflammatory (n=35) | 86.4 | 46.2 | 7.39 (1.23–44.4) | 0.020* |

**Panel B: Bootstrap Validation of Cluster 2 Odds Ratio (5,000 Iterations, Seed=42)**

| **Parameter** | **Primary Analysis (Fisher's Exact)** | **Bootstrap (5,000 Iterations)** |
| --- | --- | --- |
| Odds Ratio (point estimate) | 3.98 | 4.06 (median) |
| 95% Confidence Interval | 1.01–15.57 | 1.17–17.68 |
| Lower CI bound crosses unity (OR=1)? | Marginally (1.01) | No (1.17) |
| Interpretation | Borderline significant (p=0.047) | Non-parametric confirmation |

*Panel A: Holter-based endpoint = any AF/AFL/AT documented on the M12 Holter recording (regardless of duration or symptom burden), as opposed to the primary point-in-time endpoint. Bold indicates statistically significant CA superiority. * p<0.05 by Fisher's exact test. Panel B: Bootstrap validation used 5,000 resampling iterations with seed=42 applied to the Cluster 2 subgroup (n=67). The bootstrapped median OR (4.06) closely reproduces the Fisher's exact OR (3.98), and the lower bootstrap CI bound (1.17) does not cross unity, providing non-parametric confirmation that the Cluster 2 result is not an artefact of small-sample distribution. Abbreviations: CA = catheter ablation; LFM = lifestyle modification plus antiarrhythmic drugs; OR = odds ratio; CI = confidence interval; AF = atrial fibrillation; AFL = atrial flutter; AT = atrial tachycardia; Adv. Neurohorm./Inflammatory = Advanced Neurohormonal/Inflammatory phenotype.*
